# Predicting the Effects of Customized Corneal Cross-Linking on Corneal Geometry

**DOI:** 10.1167/iovs.66.12.51

**Published:** 2025-09-23

**Authors:** Matteo Frigelli, Miguel A. Ariza Gracia, M. Enes Aydemir, Emilio A. Torres-Netto, Farhad Hafezi, Jos Rozema, Philippe Büchler, Sabine Kling

**Affiliations:** 1 ARTORG Center for Biomedical Engineering Research, University of Bern, Switzerland; 2 ELZA Institute AG, Zürich, Switzerland; 3 Faculty of Medicine, University of Geneva, Geneva, Switzerland; 4 Department of Ophthalmology at New York University Grossman School of Medicine, New York University, New York, New York, United States; 5 Visual Optics Lab Antwerp (VOLANTIS), Faculty of Medicine and Health Sciences, University of Antwerp, Wilrijk, Belgium; 6 Department of Ophthalmology, Antwerp University Hospital, Edegem, Belgium

**Keywords:** finite element modeling, customized corneal cross-linking, optomechanical effects

## Abstract

**Purpose:**

To validate an existing finite element model (FEM) for predicting the flattening effect of corneal cross-linking (CXL) in a clinical scenario and to use this model to investigate the parameters that most influence CXL-induced flattening effects.

**Methods:**

Retrospective data were collected from two clinical cohorts, each with 20 patients receiving either standard or customized CXL. Data were collected before surgery and at the six-month follow-up. Both CXL treatments were simulated with a FEM calibrated on experimental data. Standard anterior corneal geometry indexes (e.g., sphere, cylinder), as well as the curvature changes observed at follow-up were compared to those predicted by FEM simulations.

**Results:**

At follow-up, patients who underwent customized CXL exhibited more corneal flattening compared to those who received standard CXL (Kmax-t: −2.28 ± 1.4 D vs. −0.81 ± 1.5 D; *P* < 0.001). The FEM-predicted curvature reduction in the central CXL regions showed a significant correlation with the follow-up data for both standard (*R*^2^ = 0.48, *P* < 0.01) and customized CXL (*R*^2^ = 0.59, *P* < 0.01). Compared to follow-up data, standard CXL model showed concordance correlation coefficients > 0.9 for nine corneal geometry parameters and customized CXL model for three. Sensitivity analysis demonstrated that a 3 mm Hg increase in intraocular pressure (IOP) combined with a 10% weaker keratoconus region alters flattening outcomes by up to 20%.

**Conclusions:**

Customized CXL induces a flattening of about 2 diopters in the cone region six months after surgery. The model adequately captured the curvature corrections induced by the treatment in the keratoconus cone region, but showed reduced accuracy in predicting global corneal metrics, particularly for customized CXL. The induced flattening effects depend on the IOP, keratoconus-induced biomechanical weakening, and the fluence delivered to the cone.

After its approval by the Food and Drug Administration in 2016, ultraviolet-A (UVA) corneal cross-linking (CXL) has become one of the most prevalent treatments for progressive keratoconus (KC) and other corneal ectasias, such as iatrogenic postrefractive laser surgery ectasia.^1^ CXL aims to halt the progression of these conditions by stiffening the cornea through the creation of new cross-links within the extracellular matrix.^2^ The original “Dresden protocol” for performing CXL was introduced 20 years ago.^3^ This treatment protocol involves removing the central corneal epithelium (8–9 mm optical zone) to allow the underlying stroma to be saturated with the photosensitizer riboflavin for 30 minutes. The anterior stroma is then irradiated with UVA light (365–370 nm) at an intensity of 3 mW/cm^2^ for 30 minutes, delivering a fluence of 5.4 J/cm^2^.^3^

Over the years, several variations to the Dresden protocol have been proposed to minimize patient discomfort and treatment costs during and after the intervention. Because the CXL stiffening effect is directly dependent on the amount of energy delivered to the tissue,^4^^–^^6^ one approach involves accelerating the delivery of the 5.4 J/cm^2^ fluence by increasing the intensity of UV irradiation,^7^ shortening the overall duration of the treatment, thus presumably reducing hospital waiting times and improving patient comfort.^8^ Another significant modification is retaining the epithelium (epi-on CXL) in combination with alternative methods to facilitate riboflavin absorption, such as penetration enhancers or iontophoresis,^9^ aiming to reduce the risk of early postoperative infection, pain, and haze development.^10^

The primary goal of CXL treatment is to stop the progression of KC, without aiming to correct refractive errors during the procedure. However, it has been shown that favorable refractive changes can be achieved by customizing the treatment to concentrate its effect on the weaker areas of the corneal tissue.^11^ By tailoring the procedure to the specific topography of the patient's cornea, higher energy can be directed to the KC region, thereby enhancing the CXL effect where it is most needed. In this context, the photo-therapeutic keratectomy (PTK)–assisted customized epi-on CXL protocol has been proposed. In this procedure, gradients of riboflavin and UV light are used to increase the stiffening effects in the region of the KC cone, with the combined aim of halting ectasia progression but also improving the patient's visual outcomes by enhanced corneal regularity.^12^

However, the predictability of CXL-induced refractive changes remains limited because of an incomplete understanding of how corneal mechanical alterations translate into curvature changes, and as a result these factors are currently not taken into account when planning the CXL intervention. A deeper understanding of the mechanical mechanisms of CXL is needed before changes induced by different treatment protocols can be accurately predicted and customized for each patient.^13^

A computational method such as the finite element method (FEM) offers a promising approach for understanding CXL-induced geometric changes, free from the limitations normally associated with clinical studies. Recently, our group has proposed a fully automated patient-specific FEM for predicting the flattening effects of CXL on corneal anterior curvature, starting from standard clinical topography images.^12^ The model presents two main innovations: First, it is calibrated using a combination of distinct ex vivo mechanical tests; second, it relies on the mechanical weakening observed in the KC region of the cornea measured by in vivo optical coherence elastography (OCE).

In this study, we propose a retrospective comparative FEM analysis of two cohorts of patients who received different CXL treatments: One cohort underwent customized CXL that specifically targeted the steepest apex area of the KC, whereas the other cohort received an accelerated protocol with the same fluence on the complete cornea. In particular, the present study aims to (i) assess the differences in CXL-induced curvature changes based on the location and intensity (fluence) of the CXL treatment; (ii) verify the accuracy of the previously-validated FEM in assessing the curvature changes in the patients treated with these two CXL protocols; and (iii) use the model to investigate which factors have the most influence on the flattening outcomes of the procedure.

## Material and Methods

### Data Collection

Forty KC patients who underwent CXL treatment were retrospectively enrolled, with 20 patients each from the Antwerp University Hospital, Belgium (cohort 1) and the ELZA Institute, Switzerland (cohort 2). The study was approved by the ethics committees in Antwerp (no. 17/21/254) and Zurich (no. 2021-02275), and informed consent was obtained with all data anonymized. Corneal topographies (Pentacam; Oculus, Wetzlar, Germany) were taken before treatment and at follow-up (7 ± 2 months). Data from four patients were unavailable at follow-up (one from cohort 1, three from cohort 2).

### CXL Treatments

Two different CXL methods were used in the study. Cohort 1 received a standard epi-off accelerated CXL protocol with 9 mW/cm^2^ irradiance for 10 minutes (total fluence 5.4 J/cm^2^), centered on a 9 mm diameter circular area. Cohort 2 underwent the ELZA-PTK-assisted customized epi-on (ELZA-PACE) CXL, where the epithelium was removed only over the cone region using epithelial thickness map-driven PTK. After riboflavin saturation, the cornea was irradiated with a C-eye device (EMAGine, Switzerland), delivering 14 J/cm^2^ to a 4 mm spot over the epi-off area at the tip of the cone, and 8.1 J/cm^2^ in epi-on mode over a 9 mm diameter circular area. By locally removing the epithelium and increasing the energy delivered, the procedure induces gradients of riboflavin and UV, aiming to improve vision by flattening the cone and reducing corneal irregularity.

### Patient-Specific Finite Element Modeling

The FEM used in this work has been detailed in our previous publications and is briefly summarized here.^12^^,^^14^ More information on the model and its material parameters can be found in the Supplementary Material. The FEM uses an incompressible, depth-varying hyperelastic material model in FEBio (version 4.5.0)^15^ implemented with a custom plugin.^16^ The strain energy function includes two collagen fiber families, based on the Holzapfel-Gasser-Ogden model.^17^^,^^18^ Material parameters were previously identified by fitting uniaxial tensile test data from human tissue using inverse FEM (iFEM).^14^^,^^19^ The anterior cornea was found to be 68% stiffer than the posterior,^19^ with fiber anisotropy and distribution defined from X-ray scattering and second harmonic generation microscopy.^20^^,^^21^

Patient-specific three-dimensional meshes were generated from the patients’ preoperative corneal topographies using the Python Gmsh library.^22^ Each mesh had about 150,000 second-order tetrahedral elements, with seven spanning its thickness. It was finer in the central 6 mm of the cornea and coarser in the periphery. Sliding boundary conditions were applied to the corneal-limbus junction to mimic its connection with the sclera.^23^ The KC cone area was identified in the central 8 mm of the anterior cornea, where tangential curvature exceeded the 67.5th percentile of the curvature distribution. In this region, corneal stiffness was reduced by 57%, based on in vivo OCE measurements^24^^,^^25^ from a subset of three patients.^12^ The stiffness reduction (α_*KC*_) decreased linearly from the cone center to its periphery to ensure a smooth transition between normal and KC tissue. A pre-stress algorithm was used to obtain the initial stress-free geometry, matching the patient-specific shape after pressurizing the posterior surface to a normal intraocular pressure (IOP) of 15 mm Hg for all patients.^26^^,^^27^

### Clinical Validation of CXL FEM Models

To model CXL, a stiffening factor K_CXL_ was applied to increase the stiffness of the fiber in the material model. K_CXL_ was calibrated using nanoindentation and ex vivo OCE tests on five human corneas before and after Dresden CXL (5.4 J/cm²), yielding a value of 16.3 [−] through iFEM fitting.^12^ The 16-fold increase in the fiber parameter k_1_ observed in this study corresponds to a 105% increase in the overall tangential modulus of the cornea at 15 mm Hg along the apical rise curve.

**Figure 1.**
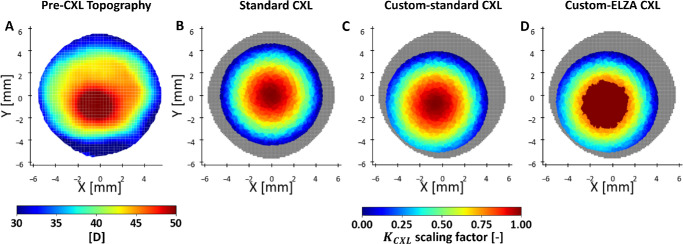
Comparison of FEM-simulated standard (**B**), custom-standard (**C**) and custom-ELZA (**D**) CXL treatments in one representative patient. (**A**) Cornea tangential curvature. (**B**, **C**, **D**) Stiffening induced in the FEM to simulate CXL.

Two CXL protocols were simulated to clinically validate the FEM (Fig. 1). In the standard CXL model (cohort 1), a radial variation of the CXL effect was modeled by smoothing the K_CXL_ factor with a Gaussian function, with the effect reducing to 10% at 90% of the radial distance from the corneal center.^28^ The CXL effect also decreased linearly with depth, reaching a value of K_CXL_=1 at 300 µm depth. In the custom-ELZA CXL model (cohort 2), the stiffening effect was centered on the KC region, with two concentric zones. The outer ring (4–9 mm diameter) used the same parameters as the standard CXL, whereas the central area (4 mm diameter) had a CXL depth of 400 µm and an increased stiffening factor (K_CXL_ELZA_ = 1.33 × K_CXL_) reflecting higher fluence in the ELZA-PACE protocol.^12^ This tuning was based on uniaxial tensile tests performed on porcine corneas showing a 33% increase in stiffening at 15 J/cm^2^ compared to the standard protocol.^6^ Clinical accuracy was assessed by comparing FEM-simulated curvature corrections with actual outcomes at follow-up for both cohorts of patients.

### FEM Analysis of CXL-Induced Geometric Changes

A third treatment, called custom-standard CXL, was modeled with standard CXL (fluence 5.4 J/cm²) centered on the KC cone instead of the corneal center (Fig. 1C) to explore the effects of treating different corneal areas on the post-operative anterior curvature. All patient models (*n* = 40) underwent each of the three CXL simulations, totaling 120 simulations. Anterior cornea geometric changes such as curvature flattening and minimum pachymetry were compared across the models.

Additionally, sensitivity analyses were performed on 10 randomly selected patients (five from each cohort) to assess how IOP and KC weakening (α_*KC*_) affected curvature outcomes. For custom-ELZA CXL, IOP was varied between 13–20 mm Hg, and α_*KC*_ between 0.3 and 0.7. For the standard and custom-standard CXL models, only one parameter was varied whereas the other remained constant (IOP = 15 mm Hg or α_*KC*_ = 0.429) to save computational time.

### Corneal Geometry Analysis

Different metrics were used to quantify the shape of the anterior cornea. To visualize corneal anterior topography and detect the KC region, tangential curvature K_tg_ (diopters) (D) was calculated using in-house Python scripts. For each node i on the corneal surface K_tg_^i^ = (1.3375 − 1)/R^i^, where R^i^ is the radius of the local best-fitting sphere within a 120 µm region around the node. Kmax-t (D) was defined as the maximal value of K_tg_. KmaxMean3 (D) was defined as the average curvature within a 3 mm region centered at the point of maximum tangential curvature Kmax-t.^29^

In addition, standard anterior corneal geometry indexes *R*_steep_ (mm), *R*_flat_ (mm) (where *R*_steep_ < *R*_flat_), sphere (D) (defined as K_flat_, where K_steep/flat_ = [1.376 − 1]/[1e – 3 · R_steep/flat_]), cylinder (D) (defined as K_steep_ − K_flat_), angle of astigmatism [°] and spherical equivalent (D) (defined as sphere + cylinder/2) were obtained on both the Pentacam topographies and FEM meshes by fitting the corneal anterior surface with a biconic surface over an 8 mm diameter region, following the approach described by Blanco-Martínez et al.^30^

### Statistical Analysis

Statistical analyses were conducted in Python (v. 3.8.8). Continuous variables were reported as means (±SD) or medians (Q1–Q3). Deltas (∆) represented the difference between post- and pretreatment values. Paired Wilcoxon tests compared pre- and post-CXL data within the same cohort, whereas independent *t*-tests compared data between cohorts. ANOVA with Tukey's post-hoc test assessed the impact of the three CXL models, and repeated measures ANOVA compared variables across four groups. A *P* value <0.05 was considered significant. The agreement between FEM-derived and clinical variables was evaluated by linear correlation, concordance correlation coefficients (CCC) and Bland-Altman analysis, expressed by bias and 95% limits of agreement (LoA).

## Results

### Clinical Differences Between the Study Cohorts

**Figure 2.**
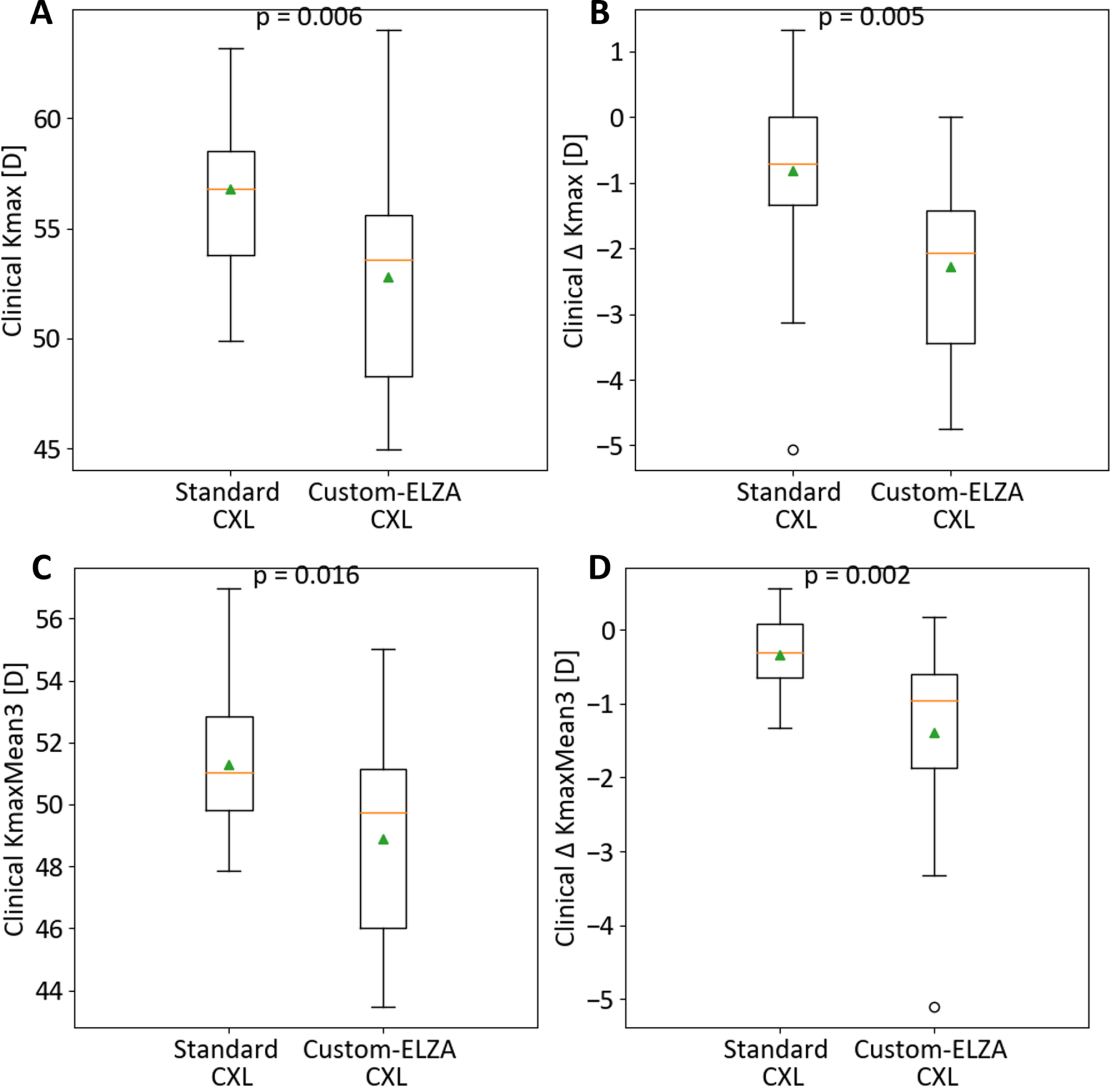
Clinical differences between cohort 1 (standard CXL) and cohort 2 (custom-ELZA PACE CXL). (**A**) Kmax-t values before CXL; (**B**) ∆ Kmax-t values after treatment; (**C**) KmaxMean3 values before CXL; (**D**) ∆ KmaxMean3 values after treatment.

Before CXL treatment, cohort 1 was significantly younger (*P* = 0.003), had higher Kmax-t (*P* = 0.006) and KmaxMean3 (*P* = 0.016) compared to cohort 2 (Table 1; Figs 2A, 2C). At follow-up, six patients (32%) in cohort 1 showed no corneal flattening (Δ Kmax-t > 0 or Δ KmaxMean3 > 0), whereas all cohort 2 patients did flatten. Cohort 2 had significantly greater reductions in Kmax-t and KmaxMean3 than cohort 1 (−2.28 ± 1.4 D vs. −0.81 ± 1.5 D, and −1.33 ± 1.3 D vs. −0.33 ± 0.6 D, *P* < 0.01) (Figs. 2B, 2D). Within the 6 mm optical zone, reductions in Kmax-t were accompanied by curvature steepening in the untreated areas, particularly in cohort 2 (0.22 ± 1.4 D vs. +2.22 ± 2.1 D; *P* < 0.01, for cohorts 1 and 2, respectively). Pachymetry also varied significantly, with cohort 1 showing minimal change (−2.8 ± 10.8 µm) and cohort 2 a larger reduction (−25.12 ± 25.3 µm; *P* < 0.01) (Table 2).

**Table 1.** Population Clinical Data Before Surgery

	Cohort 1	Cohort 2	
	(*n* = 20)	(*n* = 20)	*P* Value
Age (years)	24.5 ± 7	32.6 ± 9	**0.003**
Sex (percentage of women)	2 (10%)	8 (40%)	—
Fluence delivered to the cone (J/cm^2^)	5.4	14	—
Kmax-t (D)	56.8 ± 3.5	52.8 ± 4.9	**0.006**
KmaxMean3 (D)	51.3 ± 2.3	48.9 ± 3.4	**0.016**
Pachymetry minimum (µm)	462.8 ± 40	465.6 ± 32	0.815
R_steep_ (mm)	7.3 ± 0.5	7.4 ± 0.6	0.369
R_flat_ (mm)	7.7 ± 0.5	7.9 ± 0.5	0.423
Sphere (D)	48.9 ± 3.4	48.0 ± 3.1	0.404
Cylinder (D)	3.0 ± 1.5	2.9 ± 1.5	0.770
Angle of astigmatism	93.0° ± 70°	55.7° ± 63°	0.093
Spherical equivalent (D)	50.4 ± 3.3	49.5 ± 3.4	0.385
Missed follow-up (percentage of patients)	1 (5%)	3 (15%)	—

Statistically significant *P* values are reported in bold. The reported Kmax-t and KmaxMean3 (diopters) values were computed using the tangential curvature definition. Corneal geometry indexes were computed by fitting the cornea with a biconic surface on an 8 mm diameter area.^30^

**Table 2.** Population Clinical Data at Follow-Up and Respective Variations (Δ) Relative to the Presurgical Conditions

	Cohort 1 (*n* = 19)			Cohort 2 (*n* = 17)			
	Pre-CXL Value	Post-CXL Value	*P* Value	Pre-CXL Value	Post-CXL Value	*P* Value	*P* Value (Post-CXL Cohorts Comparison)
Age (years)	—	23.8 ± 6	—	—	32.6 ± 9	—	**0.002**
Sex (percentage of women)	—	2 (10.5%)	—	—	6 (35%)	—	—
Follow-up time (months)	—	7.9 ± 4	—	—	6.6 ± 1.1	—	0.211
Kmax-t (D)	56.8 ± 3.6	56.0 ± 3.4	**0.03**	52.4 ± 5.1	50.1 ± 4.7	**<0.001**	**<0.001**
KmaxMean3 (D)	51.4 ± 2.4	50.9 ± 2.5	**0.03**	48.6 ± 3.6	47.2 ± 3.3	**<0.001**	**<0.001**
Pachymetry minimum (µm)	463.1 ± 41	460.8 ± 42	0.09	470.1 ± 32	453.2 ± 40	**0.001**	0.710
R_steep_ (mm)	7.3 ± 0.5	7.2 ± 0.5	0.06	7.5 ± 0.5	7.6 ± 0.5	0.10	0.05
R_flat_ (mm)	7.7 ± 0.5	7.7 ± 0.5	0.14	7.9 ± 0.5	8.0 ± 0.5	0.43	0.11
Sphere (D)	48.9 ± 3.4	49.2 ± 3.5	0.15	47.5 ± 3.1	47.4 ± 3.0	0.46	0.11
Cylinder (D)	3.0 ± 1.5	3.0 ± 1.6	0.90	2.6 ± 1.3	2.4 ± 1.3	0.08	0.23
Angle of astigmatism	93.0° ± 70°	93.2° ± 71°	0.62	54.3° ± 60°	44.5° ± 52°	0.52	**0.03**
Spherical equivalent (D)	50.4 ± 3.3	50.7 ± 3.5	0.11	48.8 ± 3.2	48.6 ± 3.1	0.26	0.06
Δ Kmax-t (D)	—	−0.81 ± 1.5	—	—	−2.28 ± 1.4	—	**0.005**
Δ Kmax-t > 0D (percentage of patients)	—	6 (32%)	—	—	0	—	—
Δ Kmax-t < −3D (percentage of patients)	—	2 (10.5%)	—	—	6 (35%)	—	—
Δ KmaxMean3 (D)	—	−0.33 ± 0.6	—	—	−1.38 ± 1.3	—	**0.002**
Δ Pachymetry Minimum (µm)	—	−2.8 ± 10.8	—	—	−25.12 ± 25.3	—	**0.001**
Δ R_steep_ (mm)	—	−0.04 ± 0.1	—	—	0.03 ± 0.10	—	0.087
Δ R_flat_ (mm)	—	−0.04 ± 0.1	—	—	0.05 ± 0.1	—	**0.033**
Δ Sphere (D)	—	0.03 ± 0.8	—	—	−0.5 ± 0.7	—	0.065
Δ Cylinder (D)	—	0.49 ± 0.2	—	—	0.43 ± 0.3	—	0.496
Δ Angle of astigmatism	—	73° ± 54°	—	—	99° ± 41°	—	0.114
Δ Spherical equivalent (D)	—	0.28 ± 0.8	—	—	−0.3 ± 0.6	—	**0.045**

Statistically significant *P* values are reported in bold. The reported Kmax-t and KmaxMean3 (diopters) values were computed using the tangential curvature definition. Corneal geometry indexes were computed by fitting the cornea with a biconic surface on an 8 mm diameter area.^30^

### Finite Element Model Validation Versus Clinical Data

**Figure 3.**
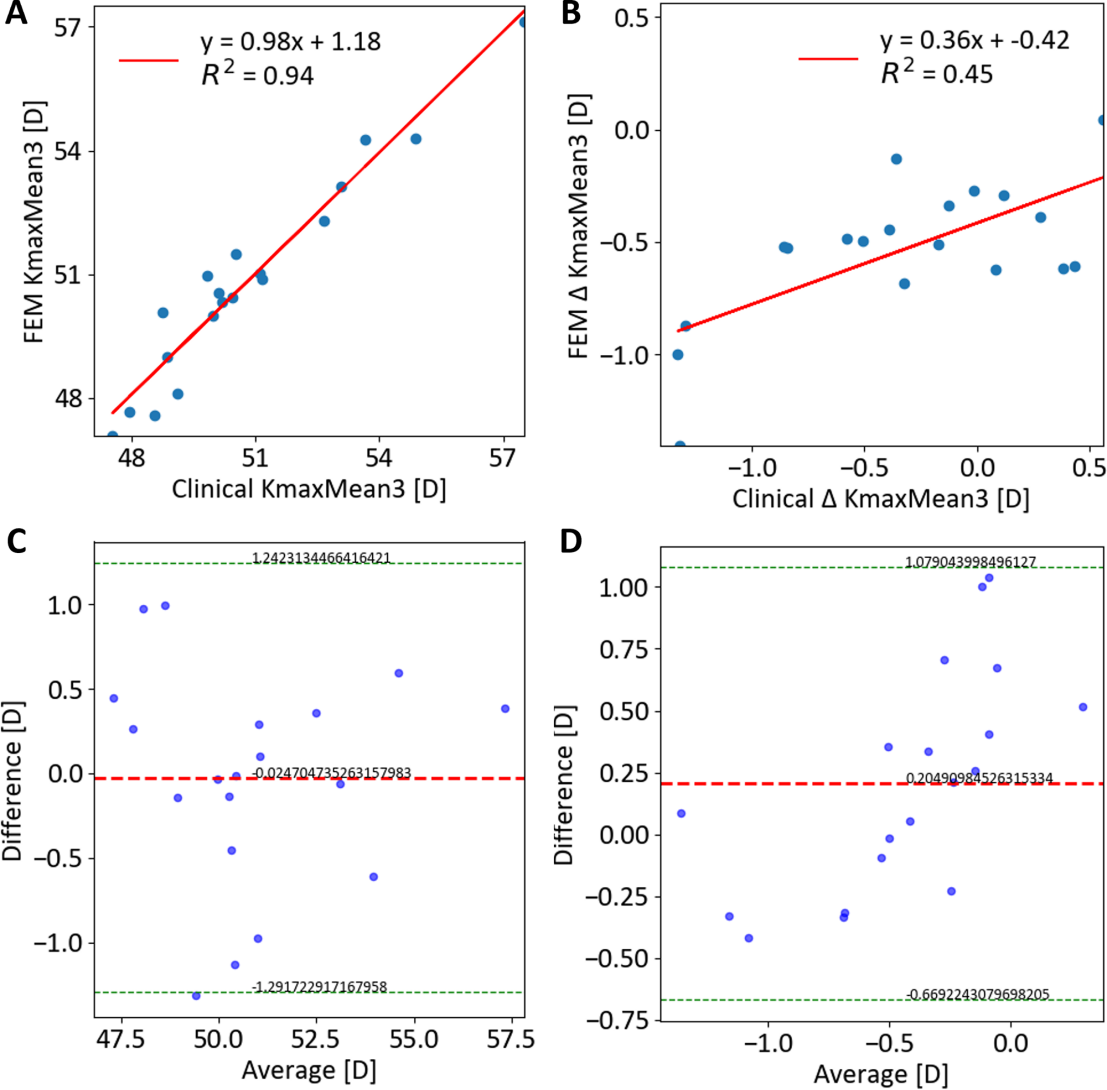
Comparison between clinical and FEM-simulated curvature values after surgery for cohort 1 (*n* = 19). (**A**) Correlation of KmaxMean3; (**B**) Correlation of ∆ KmaxMean3; (**C**) Bland–Altman plot of KmaxMean3; (**D**) Bland–Altman plot of ∆ KmaxMean3.

**Figure 4.**
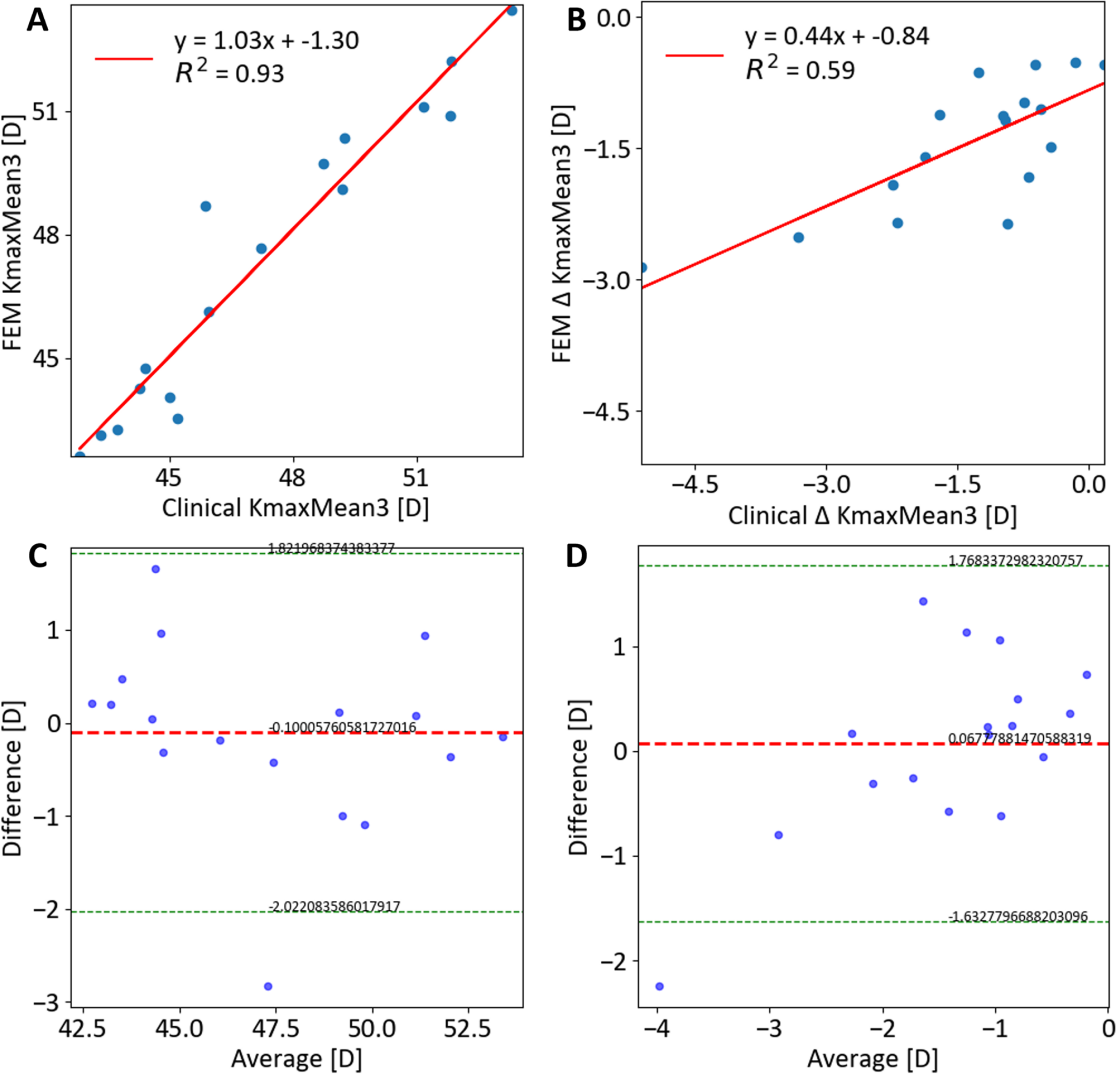
Comparison between clinical and FEM-simulated curvature values after surgery for cohort 2 (*n* = 17). (**A**) Correlation of KmaxMean3; (**B**) Correlation of ∆ KmaxMean3; (**C**) Bland–Altman plot of KmaxMean3; (**D**) Bland–Altman plot of ∆ KmaxMean3.

**Figure 5.**
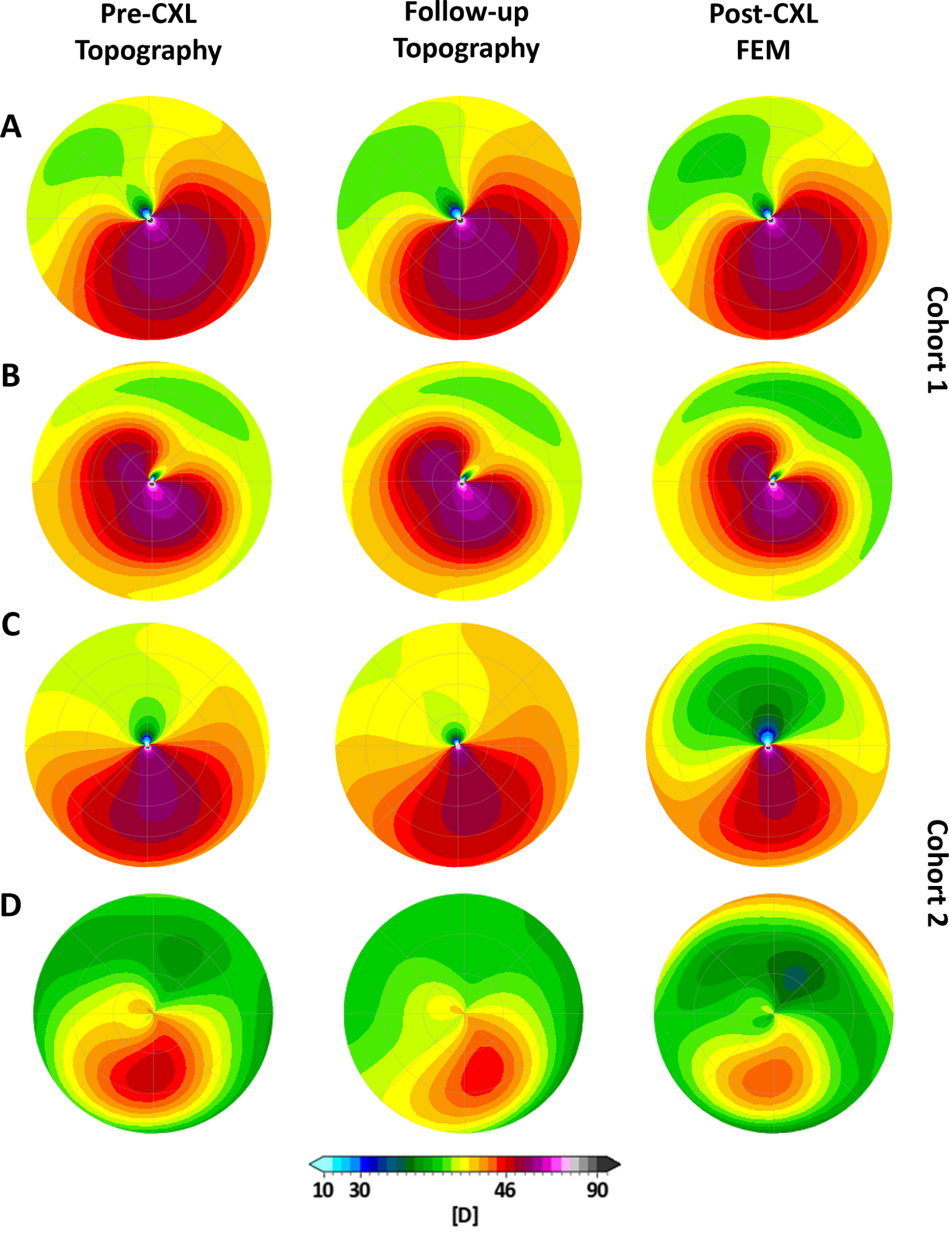
Tangential curvature maps (**D**) obtained on a 6 mm diameter optical zone for four representative patients (one per row). The left column represents the Pentacam topography before CXL, the central column the Pentacam topography at follow-up, and the right column the corresponding post-CXL FEM prediction. (**A**, **B**) Patients from cohort 1 who received standard accelerated CXL. (**C**, **D**) Patients from cohort 2 who received customized epi-on (ELZA-PACE) CXL. Post-CXL topographies show anterior corneal steepening in the areas unaffected by KC.

No significant differences in curvature or minimal thickness (*P* > 0.1) were found between pre-operative clinical data and the pre-CXL model. Strong correlations were observed for KmaxMean3 (*R*^2^ = 0.99, *P* < 0.01) and minimum pachymetry (*R*^2^ = 1.00, *P* < 0.01) in both cohorts (Supplementary Figs. S1, S2). Data from one patient in cohort 1 and three patients in cohort 2 were unavailable at follow-up and were therefore excluded from the post-CXL correlations reported in this paragraph. Post-CXL, significant differences in minimum pachymetry variations (*P* < 0.012) were noted, with the model showing a slight thickness increase in cohort 1 (4.6 ± 0.3 µm) and cohort 2 (8.2 ± 0.4 µm). Significant correlations between clinical and FEM post-CXL KmaxMean3 were observed for both cohorts (*R*^2^ = 0.94 and 0.93, *P* < 0.01) (Table 3; Figs. 3A, 4A). Bland-Altman analyses showed minimal bias (−0.02 D and −0.1 D) (Table 3; Figs. 3C, 4C). No significant differences in curvature variations were found between the model and both cohorts (*P* > 0.08). The model accurately predicted reductions in KmaxMean3, correlating well with clinical data of both cohorts (*R*^2^ = 0.45 and 0.59, *P* < 0.01) (Figs. 3B–D, 4B–D), and captured post-CXL effects for both treatment methods, including curvature reduction in the KC region. The model reported an increase in curvature in the KC-free regions within a 6 mm optical zone in cohort 2 but not in cohort 1 (1.18 ± 0.7 D vs −0.71 ± 0.5 D, *P* < 0.01, for cohort 2 and 1, respectively) (Fig. 5).

**Table 3.** Correlation Between Clinical and FEM-Simulated Anterior Cornea Geometry After Surgery

	Cohort 1 – Standard CXL (*n* = 19)						Cohort 2 – Custom-ELZA PACE CXL (*n* = 17)					
	Clinical Post-CXL	FEM Post-CXL	*t*-Test *P* Value	Pearson *R*^2^	CCC	Bias (95% LoA)	Clinical Post-CXL	FEM Post-CXL	*t*-Test *P* Value	Pearson *R*^2^	CCC	Bias (95% LoA)
KmaxMean3 (D)	50.9 ± 2.5	50.9 ± 2.5	0.984	0.937	0.968	−0.02 [−1.3, 1.2]	47.2 ± 3.3	47.3 ± 3.5	0.818	0.927	0.96	−0.1 [−2.0, 1.8]
Pachymetry Minimum (µm)	460.8 ± 42	470.0 ± 39	**0.018**	0.900	0.927	−8.4 [−34.0, 17.1]	453.2 ± 40	478.1 ± 34	**<0.001**	0.763	0.707	−25.2 [−66.1, 15.6]
Kmax-t (D)	56.0 ± 3.4	56.4 ± 3.9	0.352	0.840	0.905	−0.5 [−3.6, 2.6]	50.1 ± 4.7	50.6 ± 6.3	0.597	0.897	0.911	−0.4 [−5.2, 4.3]
R_steep_ (mm)	7.2 ± 0.5	7.3 ± 0.5	0.073	0.902	0.927	−0.1 [−0.4, 0.3]	7.6 ± 0.5	7.9 ± 0.6	**0.002**	0.773	0.776	−0.3 [−0.9, 0.3]
R_flat_ (mm)	7.7 ± 0.5	7.9 ± 0.5	**0.003**	0.928	0.932	−0.1 [−0.4, 0.2]	8.0 ± 0.5	8.3 ± 0.5	**<0.001**	0.713	0.634	−0.4 [−0.9, 0.1]
Sphere (D)	49.2 ± 3.5	48.0 ± 3.1	**0.005**	0.918	0.932	0.7 [−1.1, 2.5]	47.4 ± 3.0	45.4 ± 2.6	**<0.001**	0.740	0.68	2.2 [−0.6, 5.1]
Cylinder (D)	3.0 ± 1.6	3.4 ± 1.8	**0.023**	0.870	0.913	−0.3 [−1.7, 1.0]	2.4 ± 1.3	2.7 ± 1.4	**0.001**	0.876	0.893	−0.4 [−1.4, 0.6]
Angle of astigmatism	93.2° ± 71°	96.9° ± 68°	0.312	0.993	0.996	1.2 [−11, 13]	44.5° ± 52°	64.5° ± 64.3°	0.782	0.363	0.565	−18.6 [−127, 89]
Spherical equivalent (D)	50.7 ± 3.5	49.7 ± 3.2	**0.012**	0.911	0.935	0.5 [−1.5, 2.4]	48.6 ± 3.1	46.8 ± 3.0	**<0.001**	0.767	0.722	2.1 [−1.0, 5.1]

Paired comparisons between clinical and FEM-derived values within the same cohort of patients, Pearson correlation coefficient, CCC and Bland-Altman bias are reported for the different variables. Statistically significant *P* values are reported in bold. The reported Kmax-t and KmaxMean3 (diopters) values were computed using the tangential curvature definition. Corneal geometry indexes were computed by fitting the cornea with a biconic surface on an 8 mm diameter area.^30^

### FEM Insights of CXL-Induced Geometric Changes

**Figure 6.**
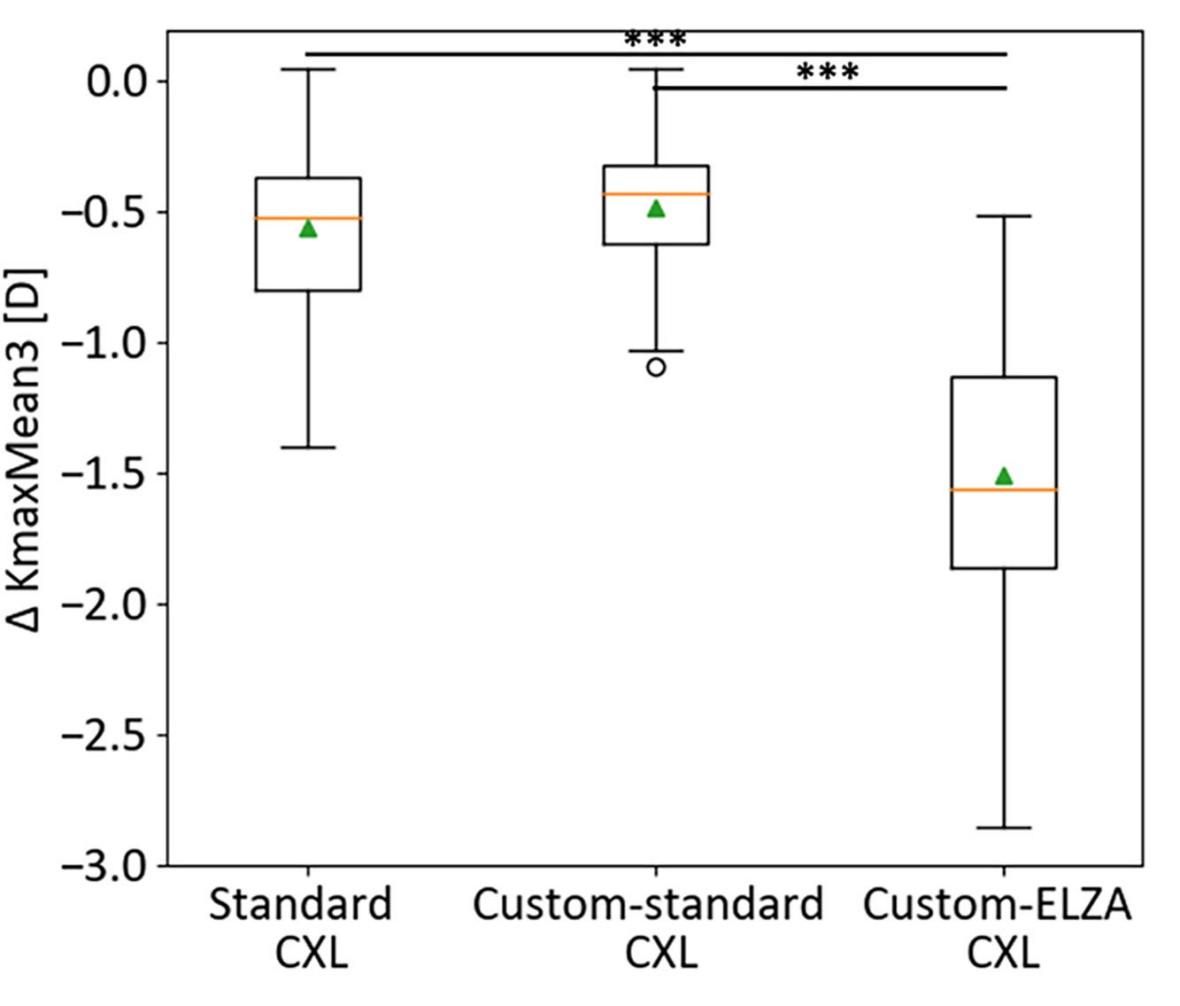
Anterior cornea curvature change (expressed as ∆ KmaxMean3) differences between the three FEM-simulated CXL treatments for the entire study cohort (*n* = 40). Significant differences in the post-hoc analysis are highlighted. ***P ≤ 0.001.

**Figure 7.**
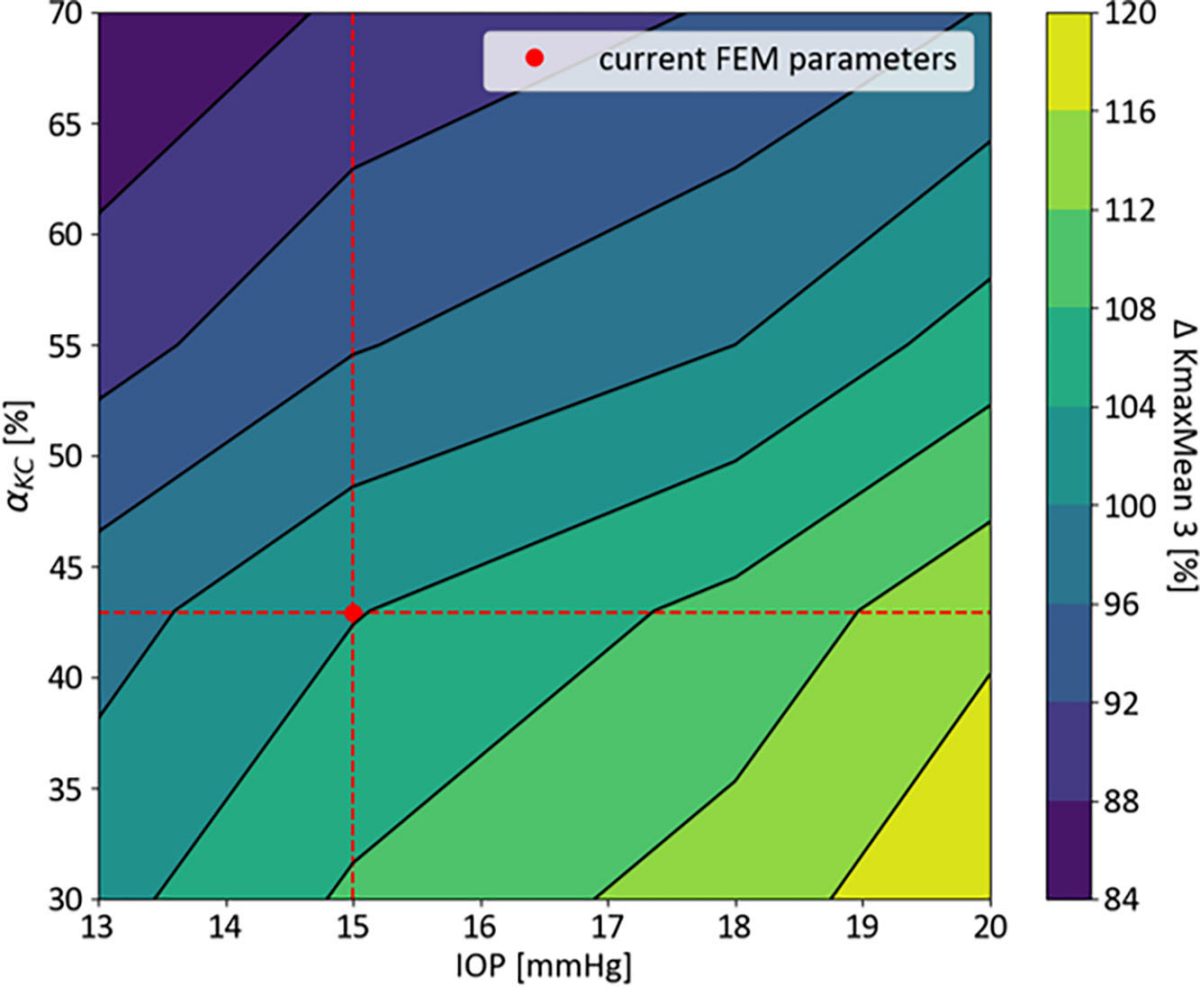
Sensitivity analysis on IOP and KC-induced weakening performed on a subset of patients randomly selected from both cohorts (*n* = 10). Custom-ELZA protocol only was simulated in this analysis. The color scale corresponds to percentage variations w.r.t. ∆ KmaxMean3 value obtained with IOP = 15 mm Hg and α_*KC*_ = 0.429, indicated as the red dot in the graph.

ANOVA analysis of the three FEM-simulated CXL methods in the study cohort (*n* = 40) showed significant differences in curvature corrections (*P* < 0.001; Table 4). Custom-ELZA CXL resulted in significantly greater reductions in KmaxMean3 (−1.51 ± 0.6 D) compared to standard CXL (−0.56 ± 0.3 D) and custom-standard CXL (−0.49 ± 0.3 D) (*P* < 0.001; Fig. 6). It also led to a higher increase in minimum pachymetry (*P* < 0.001). No significant differences were found between standard and custom-standard CXL for KmaxMean3 or minimal thickness changes (Table 4). Sensitivity analysis of custom-ELZA CXL showed that changes in IOP and KC-induced weakening could cause up to ±20% variation in anterior curvature flattening, with high IOP and weak corneas leading to greater corrections. The standard CXL and custom-standard CXL showed similar trends (Fig. 7; Table 5).

**Table 4.** FEM-Derived Variations of Anterior Cornea Geometry Induced by the Surgery on the Entire Study Cohort (*n* = 40)

	Standard CXL (*n* = 40)	Custom-Standard CXL (*n* = 40)	Custom-ELZA CXL (*n* = 40)	ANOVA *P* Value	Standard Vs. Custom-Standard	Standard Vs. Custom-ELZA	Custom-Standard Vs. Custom-ELZA
Δ KmaxMean3 (D)	−0.56 ± 0.3	−0.49 ± 0.3	−1.51 ± 0.6	**<0.01**	0.698	**<0.001**	**<0.001**
Δ Pachymetry Minimum (µm)	4.7 ± 0.3	4.8 ± 0.3	8.2 ± 0.5	**<0.01**	0.706	**<0.001**	**<0.001**
Δ Kmax-t (D)	−0.60 ± 0.4	−0.61 ± 0.4	−1.64 ± 1.1	**<0.01**	0.999	**<0.001**	**<0.001**
Δ R_steep_ (mm)	0.16 ± 0.05	0.14 ± 0.03	0.39 ± 0.13	**<0.01**	0.695	**<0.001**	**<0.001**
Δ R_flat_ (mm)	0.19 ± 0.05	0.17 ± 0.04	0.44 ± 0.15	**<0.01**	0.800	**<0.001**	**<0.001**
Δ Sphere (D)	−1.15 ± 0.2	−1.1 ± 0.15	−2.75 ± 0.74	**<0.01**	0.873	**<0.001**	**<0.001**
Δ Cylinder (D)	0.09 ± 0.05	0.16 ± 0.09	0.20 ± 0.22	**<0.01**	0.147	**0.006**	0.413
Δ Angle of astigmatism	84.5° ± 62°	81.6° ± 73°	89.0° ± 61°	>0.1	0.982	0.955	0.886
Δ Spherical equivalent (D)	−1.11 ± 0.2	−1.02 ± 0.1	−2.65 ± 0.8	**<0.01**	0.749	**<0.001**	**<0.001**

ANOVA *P* values and post-hoc analysis *P* values are reported. Statistically significant *P* values are reported in bold. The reported ∆ Kmax-t and ∆ KmaxMean3 (diopters) values were computed using the tangential curvature definition. Corneal geometry indexes were computed by fitting the cornea with a biconic surface on an 8 mm diameter area.^30^

**Table 5.** Sensitivity Analysis Performed on a Subset of Patients Randomly Selected From Both Cohorts (*n* = 10)

	IOP (mm Hg)					α_*KC*_ [–]				
	13	15	18	20	*P* Value	0.3	0.429	0.55	0.7	*P* Value
Standard CXL (*n* = 10)	−0.51 ± 0.2	−0.56 ± 0.2	−0.62 ± 0.2	−0.67 ± 0.2	**<0.001**	−0.59 ± 0.2	−0.56 ± 0.2	−0.53 ± 0.2	−0.50 ± 0.2	**<0.001**
Custom-standard CXL (*n* = 10)	−0.46 ± 0.2	−0.52 ± 0.2	−0.58 ± 0.2	−0.63 ± 0.2	**<0.001**	−0.55 ± 0.2	−0.52 ± 0.2	−0.48 ± 0.2	−0.45 ± 0.2	**<0.001**
Custom-ELZA CXL (*n* = 10)	−1.72 ± 0.3	−1.86 ± 0.3	−2.03 ± 0.3	−2.12 ± 0.3	**<0.001**	−1.97 ± 0.3	−1.86 ± 0.3	−1.78 ± 0.3	−1.68 ± 0.3	**<0.001**

Δ KmaxMean3 (D) values are reported for each of the FEM analysis. Statistically significant repeated measures ANOVA *P* values are reported in bold. IOP = 15 mm Hg and α_*KC*_ = 0.429 were adopted when varying α_*KC*_ and IOP values, respectively. The reported ∆ KmaxMean3 (diopters) values were computed using the tangential curvature definition.

## Discussion

The location and degree of CXL-induced corneal stiffening affect tissue differently in terms of refractive changes, which are often overlooked in preoperative planning. This study analyzed corneal anterior curvature changes from two CXL protocols, showing that customized ELZA-PACE-CXL produces a stronger corneal flattening effect than standard CXL. A validated patient-specific FEM accurately predicted post-operative topographies (*R*^2^ > 0.92) and curvature changes in both cohorts. The model also showed that targeting the cone alone is insufficient to produce substantial flattening but must be combined with higher UV fluence.

Our clinical results showed a Kmax-t reduction of −0.81 ± 1.5 D six months after accelerated CXL, which is similar to the reduction reported by Seiler et al. (−0.9 ± 1.3 D) at one year in 19 patients treated with the same protocol.^11^ Larger studies by Koller et al.^31^ (*n* = 151) and Toprak et al.^32^ (*n* = 96) found comparable reductions of 0.89 ± 1.49 D and 0.98 D, respectively, at one year. Hersh et al.^33^ reported a higher reduction of 1.7 ± 3.9 D after standard CXL, but 30% of their patients had post-LASIK ectasia, limiting comparability.

The amount of postoperative flattening after standard CXL is linked to the preoperative location of the cone, with greater flattening in eyes where KC is within the central 3 mm of the cornea, where the irradiance is higher. Roy and Dupps^28^ also found that CXL outcomes, such as corneal flattening and reduction in higher-order aberrations, depend on how stiffening is distributed relative to the cone. It is known that peripheral areas receive less UVA irradiation during treatment,^34^ causing less stiffening toward the periphery and smaller curvature changes for standard CXL. Therefore customized CXL targets UVA irradiation on the KC region, aiming to achieve greater corneal flattening. Our results show an average 2.3 ± 1.4 D reduction in Kmax-t at six months after surgery, with 35% of patients experiencing over 3 D reduction after ELZA-PACE CXL. This is slightly higher than reported by Seiler et al.^11^ (−1.7 ± 2.0 D) at one year, likely because of their lower fluence (10 J/cm^2^ vs 14 J/cm^2^). Smaller changes were reported by Cassagne et al. (−1.29 ± 2.44 D) and Nordström et al. (−1.29 ± 2.44 D) with customized CXL delivering variable fluences (up to 15 J/cm^2^) pulsed at 1-second intervals, using the Avedro KXL II system.^35^^,^^36^ The effectiveness of customized CXL-derived curvature changes seems to be linked to the fluence delivered to the cone. In this context, the ELZA-PACE CXL method introduces riboflavin and UV light gradients delivering high energy to specific corneal areas. This method stabilizes the cornea and reduces its asymmetry, as shown by the steepening observed in the areas unaffected by KC, providing both disease control and improved visual outcomes. Clinical data collected in this study demonstrated that KmaxMean3 better describes KC regression when compared to conventional global metrics of corneal geometry, confirming previous findings.^29^ Accordingly, this metric was employed as the benchmark in the FEM computations.

Patients from cohort 1 were significantly younger than patients from cohort 2 and displayed a higher corneal curvature, indicating more advanced KC before the treatment. These differences might suggest that patients from cohort 1 had softer corneas compared to patients from cohort 2, because of both the younger age and more pronounced KC presence. The clinical differences reported in this study are therefore even more significant when considering that the curvature corrections obtained with ELZA-PACE CXL were performed on stiffer corneas.

The FEM in this study accurately represented preoperative corneal thickness and curvature and matched postoperative KmaxMean3 variations. It was able to replicate the steepening observed in areas unaffected by KC in patients who underwent customized CXL, thereby reducing corneal asymmetry. The model showed better agreement with patients who underwent custom-ELZA PACE-CXL (*R*^2^ = 0.59) compared to standard CXL (*R*^2^ = 0.45) when analyzing KmaxMean3 variations. Lower correlation in cohort 1 may be due to *n* = 6 patients who showed no flattening or steepening in the regions treated with CXL, which a purely mechanical FEM cannot capture. Excluding these patients improved the model's predictive accuracy (*R*^2^ = 0.66; Supplementary Fig. S3). The increased pachymetry predicted by the model post-CXL aligns with the expected thickening of a stiffer cornea compared to a softer one under the same IOP, but it did not match clinical observations. The CXL model adopted in this study did not take into account the decreased spacing between lamellae observed in the cornea when additional crosslinks are induced in the tissue.^37^ This limitation, along with the limited resolution of the model throughout the thickness direction (each element spanning ∼70 µm), might explain the poor matching between FEM and clinical data when comparing postoperative pachymetry values.

The CXL stiffening effect in this model was radially attenuated using a Gaussian function, reaching no stiffening at the edge of the irradiated region. This approach only approximates clinical UVA light devices, which show smaller (about 10%) intensity drop at the edges.^28^ However, modeling this drop would create sharp stiffness transitions at the boundary, with elements at the 9 mm diameter region having an elastic modulus 16 times stiffer than neighboring elements, potentially impacting FEM analysis and numerical convergence. Therefore the model simulated greater radial attenuation of UVA light to induce smoother transitions at the peripheral CXL areas. This approach is based on the variable-intensity protocols proposed by Roy and Dupps^28^ who replicated the measured radial attenuation of an experimental UVA light source. In their in silico study, they demonstrated that this approach produced a Kmax-t reduction nearly 1 D greater than simulations with less beam attenuation at the edges. Additionally, the characteristics of each UVA source may vary, as clinical UVA sources are known to produce less intensity reduction with increasing beam radius compared to experimental devices.^28^ Although the impact of radial attenuation must be considered, it was applied consistently across all CXL protocols in this study for accurate internal comparisons.

One of the challenges in customized CXL is accurately centering the treatment on the KC cone. Common methods center on either the highest keratometry or thinnest pachymetry points,^38^ as used in ELZA-PACE CXL. Some suggest centering on the posterior float maxima, because pachymetry and corneal curvature can be affected by epithelial thickness and tear film.^11^ Ideally, the cone should be identified as the cornea's weakest point, particularly in early KC stages when geometrical modifications are not visible,^39^ but in vivo stiffness mapping is not yet clinically available. Despite promising results,^40^^,^^41^ air-puff technologies such as Corvis ST measure corneal mechanics only globally, thus not allowing for a localized investigation. Technologies like Brillouin microscopy and OCE offer higher spatial resolution for in vivo corneal biomechanics.^24^^,^^42^^–^^44^ Not only do these technologies allow for more detailed spatial localization of the cone, but they could also improve customized CXL planning by quantifying the patient-specific mechanical properties degradation induced by KC. In fact, as demonstrated by the sensitivity analysis performed in this study, treating weaker KC regions leads to a 10% to 15% increase in the predicted flattening outcome, with this effect being more pronounced (20%) when IOP values exceed 18 mm Hg. This model incorporates preoperative in vivo OCE data from three individuals, marking a step toward personalized computational tools that consider both geometry and mechanical properties, improving CXL outcome predictions.

The model presented in this study was created using in vivo OCE data and calibrated with independent ex vivo data testing both tensile and compressive tissue behavior.^12^^,^^14^^,^^19^ It is the first independently calibrated model used to simulate individual CXL geometric effects. Such a model could guide preoperative decisions on the best location, size, and energy intensity for patient-specific corrections. The FEM analysis found that centering treatment on the KC cone without increasing energy delivers similar results as standard fluences. To achieve significant corneal flattening in customized CXL, higher energy delivery to the cone is recommended.

In addition to the specific application of ectasia treatment, customized CXL has been proposed for treating low to moderate refractive errors in healthy individuals because of its cost-effectiveness and low invasiveness.^45^^,^^46^ However, it hasn't gained wide adoption because of less predictable outcomes compared to conventional refractive interventions,^47^ and the continuous flattening shown by some patients over years after surgery was performed.^48^ In this context, the present model could help improve the predictability of customized CXL treatments.

A limitation of this study is the relatively short follow-up period, the small number of patients, and their age difference, which limited statistical analysis. Additionally, IOP measurements were unavailable because of the inaccuracy of tonometry in KC,^49^ so a standard value of 15 mm Hg was used. This overlooks individual variations, as the sensitivity analysis showed curvature changes could vary by 20% with different IOP levels in a physiologic range. Similarly, corneal mechanical properties were taken from ex vivo tests on older donor corneas, while the distribution of collagen fibers was based on previous literature reports. The lack of patient-specific data may contribute to certain inaccuracies in prediction. The model doesn't include epithelium or stromal dynamic remodeling effects but only investigates a “snapshot” of the tissue status six months after CXL. It was initially calibrated using experimental Dresden protocol data and then adjusted for higher energy levels based on data collected on porcine corneas,^6^ which may explain some of the observed prediction inaccuracies for the higher energy protocol. While the model captured ELZA-PACE CXL–induced changes in the conical region, its accuracy was reduced in representing global corneal metrics, particularly with the customized CXL approach. Future work will aim to enhance the model's performance in predicting the full post-CXL corneal shape. The present FEM should be recalibrated if applied to investigate different CXL protocols, as variations in irradiation time, soaking period, and riboflavin osmolarity can lead to different stiffening, therefore geometric, effects.

Despite these limitations, the study demonstrated that: (i) ELZA-PACE CXL leads to significantly greater corneal flattening in KC patients 6 months post-surgery compared to standard CXL; (ii) FEM is a valuable tool for predicting patient-specific curvature changes in customized CXL; (iii) higher anterior corneal flattening over the cone and reduction in asymmetry is achieved by increasing mechanical stiffening with higher fluences focused on the KC cone. FEM-driven analyses can help identify patient-specific factors that influence CXL outcomes, improving treatment planning.

## Supplementary Material

Supplement 1
