# Supplementary material for: Predicting the Effects of Customized Corneal Cross-Linking on Corneal Geometry
Source: Invest Ophthalmol Vis Sci. 2025 Sep 23;66(12):51. doi: 10.1167/iovs.66.12.51 (PMC12468094; doi:10.1167/iovs.66.12.51)
Supplement: Supplement 1 [file iovs-66-12-51_s001.pdf]

Predicting the Effects of Customized Corneal Cross-Linking on Corneal Geometry

Matteo Frigelli[1], Miguel A. Ariza Gracia[1], M. Enes Aydemir[2], Emilio A. Torres-Netto[2], Farhad Hafezi[2,3,4], Jos Rozema[5,6], Philippe Büchler[1], Sabine Kling[1]

[1] ARTORG Center for Biomedical Engineering Research, University of Bern, Switzerland

[2] ELZA Institute AG, Zürich, Switzerland

[3] Faculty of Medicine, University of Geneva, Geneva, Switzerland

[4] Department of Ophthalmology at New York University Grossman School of Medicine, New York University, New York, USA

[5] Visual Optics Lab Antwerp (VOLANTIS), Faculty of Medicine and Health Sciences, University of Antwerp, Wilrijk, Belgium

[6] Department of Ophthalmology, Antwerp University Hospital, Edegem, Belgium.

Corresponding Author:
Sabine Kling
ARTORG Center for Biomedical Engineering Research
University of Bern
Freiburgstrasse 3
3010 Bern, Switzerland
sabine.kling@unibe.ch

Funding: This work received funding from the European Union's HORIZON 2020 research and innovation programme under grant agreement No 956720.

Supplementary Materials

Finite element model formulation

An exhaustive description of the hyperleastic material model reported in the method section is here presented. Two dispersed collagen fiber families were included in the strain-energy function reported in Eq. (1), using a weighted integration of the Holzapfel-Gasser-Ogden (HGO) model over the unit sphere, with 350 integration points. $\bar{I}_1$ and $\bar{I}_4$ are invariants of the isochoric Cauchy-Green strain tensor. $\bar{C} = J^{2/3}\boldsymbol{F}^T\boldsymbol{F}$, $\boldsymbol{F}$ is the deformation gradient tensor, and $J = \det(\boldsymbol{F})$. The Macaulay bracket operator, denoted as < • >, is employed to account for the fact that collagen fibers only contribute to the overall mechanical response when they are in a state of tension:

$$\begin{aligned} \bar{I}_1 &= \mathrm{Tr}\ \bar{C} \\ \bar{I}_4 &= (\mathbf{a} \otimes \mathbf{a}) : \bar{C} \\ \langle \bar{E} \rangle &= \begin{cases} \bar{E} & \text{if } \bar{E} > 0 \\ 0 & \text{if } \bar{E} \leq 0 \end{cases} \end{aligned} \tag{S.1}$$

The general fiber direction in spherical coordinates, $\boldsymbol{a}$, is described as:

$$\begin{gathered} \boldsymbol{a}\ (\varphi, \theta) = cos\varphi\ sin\theta\ \boldsymbol{e_1} + sin\varphi\ sin\theta\ \boldsymbol{e_2} + cos\theta \boldsymbol{e_3} \\ 0 \leq \phi \leq 2\pi, 0 \leq \theta \leq \pi \end{gathered} \tag{S.2}$$

The angular density of the fiber distribution ρ was decomposed as a product of the in-plane and out-of-plane distributions ρ_{in} and ρ_{out}:

$$\begin{aligned} \rho(\varphi, \theta) &= \rho_{op}(\theta)\rho_{ip}(\varphi) \\ \rho_{op}(\theta) &= 2\sqrt{\frac{2b}{\pi}} \frac{\exp[-2b\cos^2\ \theta]}{\mathrm{erf}\,(\sqrt{2b})}, 0 \leq \theta \leq \pi \\ \rho_{ip}(\varphi) &= \frac{\exp\ [a\cos\ (2\varphi)]}{I_0(a)}, 0 \leq \varphi \leq 2\pi \end{aligned} \tag{S.3}$$

Where, I_0 is the modified Bessel function of the first kind of order 0. The fiber distribution parameters a and b were defined based on x-ray scattering and second harmonic generation data. While b=2.5 was kept constant along the corneal curvature, the parameter a went from a=0 (isotropic in-plane dispersion) in the anterior part to a=5 (aligned fibers direction) in the posterior part of the cornea.

In order to model the weaking effect of KC and the stiffening effect of CXL on the tissue, the parameter k_1 related to the stiffness of the fiber component of the model was multiplied by a weaking factor α_{KC} and a stiffening factor K_{CXL}, respectively. Both values showed spatial dependence, reaching their maximum at the center of the KC and CXL regions and gradually reaching 1 (no modifications to k_1 induced) towards the periphery. The parameters adopted in the FEM are summarized in Table S1.

k [MPa]	C_{10} [kPa]	k_1^{ant} [MPa]	k_2 [-]	f_c [-]	a_{ant} [-]	a_{post} [-]	b [-]	α_{KC} [-]
10^3	25	4.56	18.9	0.02	0	5	2.5	0.429

Table S1: Material parameters adopted in the pre-CXL FEM.

Pre-CXL FEM accuracy

The comparison between the FEM geometries and the pre-operative clinical data geometries is here reported both in terms of corneal curvature and thickness.

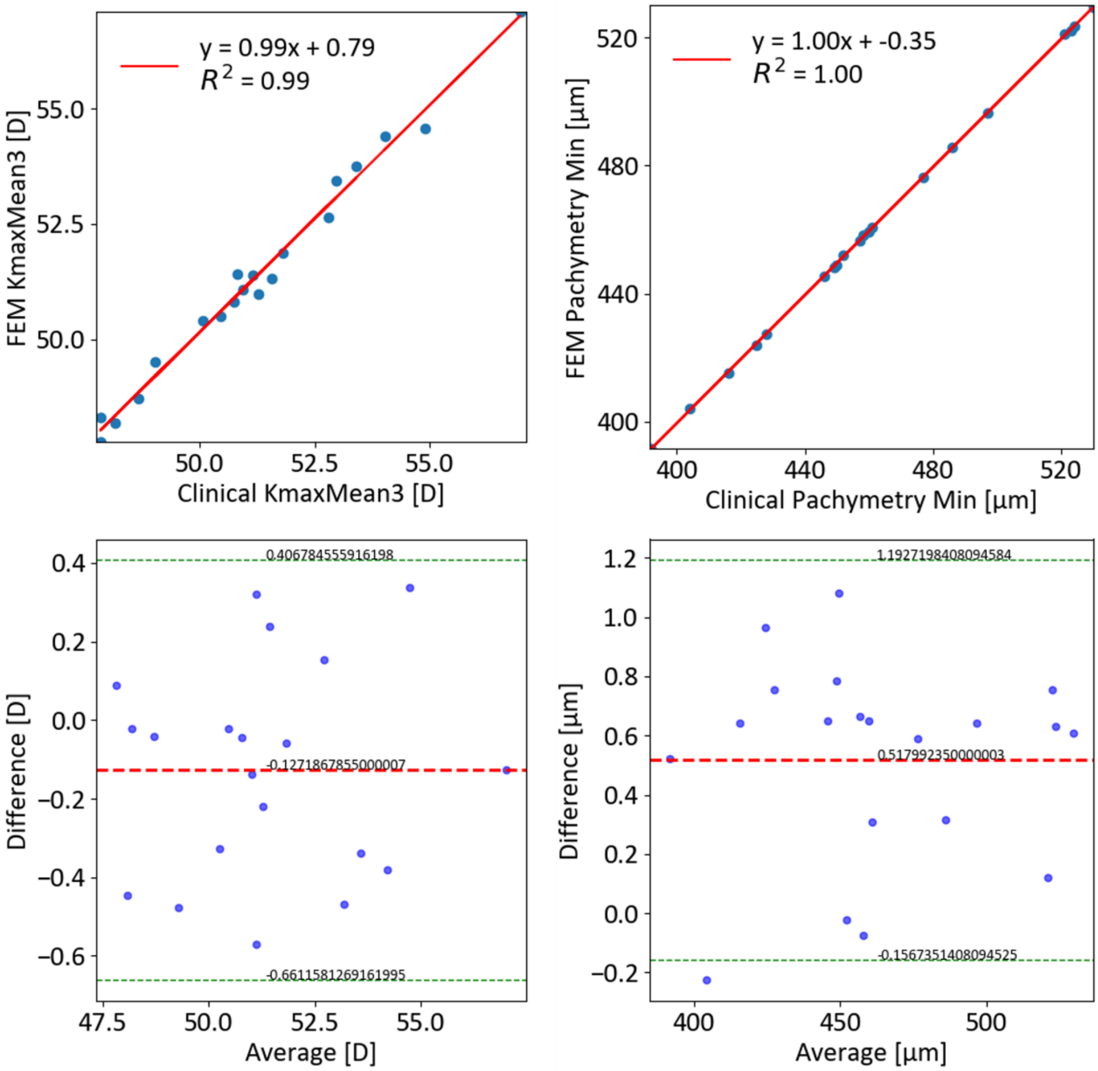

Figure S1: Comparison between clinical and FEM-simulated KmaxMean3 (left column) and min pachymetry (right column) values before surgery for cohort 1 (n=20). Correlation (top row) and Bland-Altman plot (bottom row).

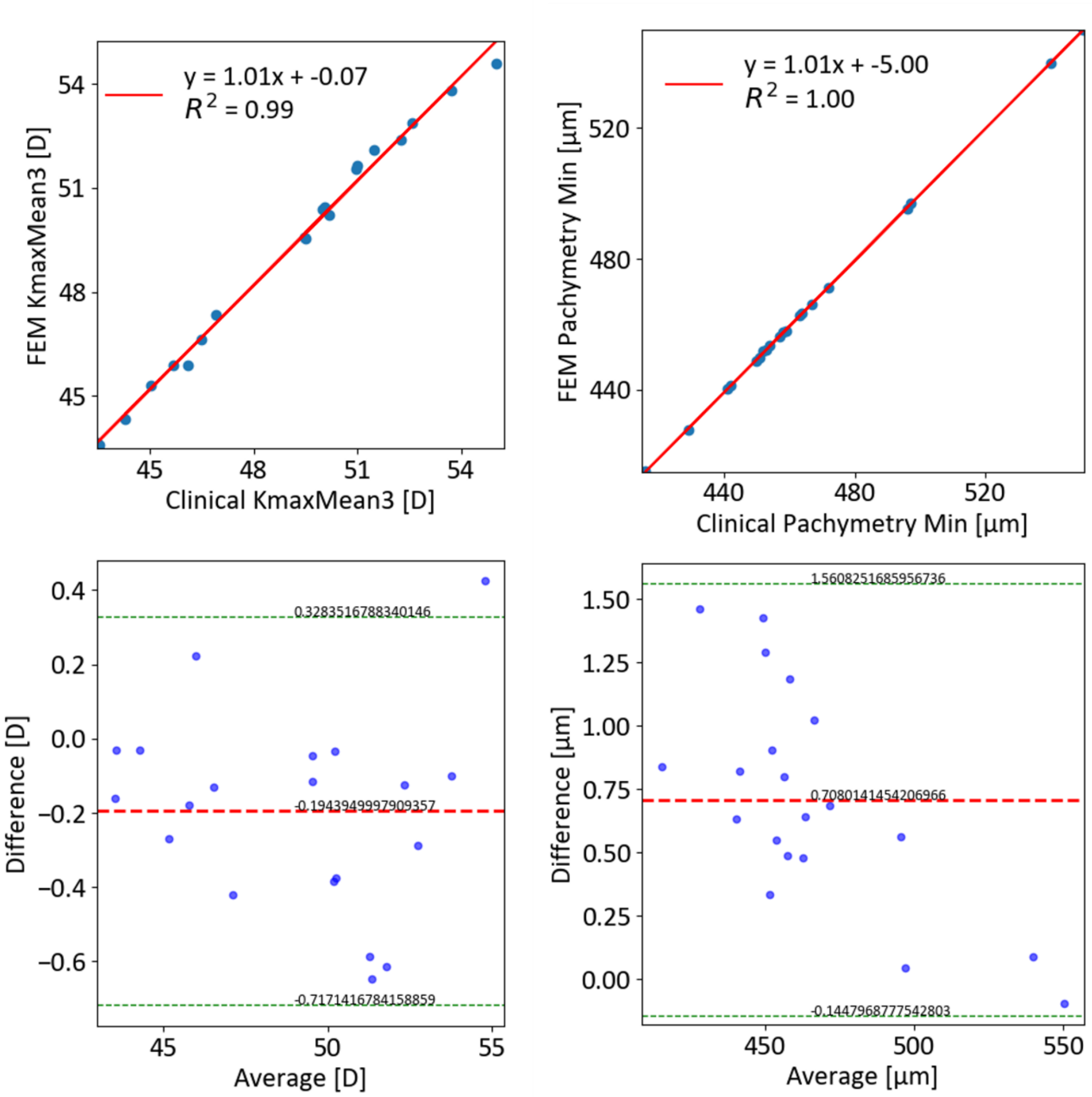

Figure S2: Comparison between clinical and FEM-simulated KmaxMean3 (left column) and min pachymetry (right column) values before surgery for cohort 2 (n=20). Correlation (top row) and Bland-Altman plot (bottom row).

Additional FEM insights

32% (n=6) of patients from cohort 1 didn't undergo any flattening of the anterior cornea (Δ KmaxMean3>0) at the 6-month follow-up, while no patients from cohort 2 did. When trying to predict CXL-induced curvature changes only in patients from cohort 1 who underwent anterior cornea flattening, the model proved to be a better predictor of CXL-induced geometric effects.

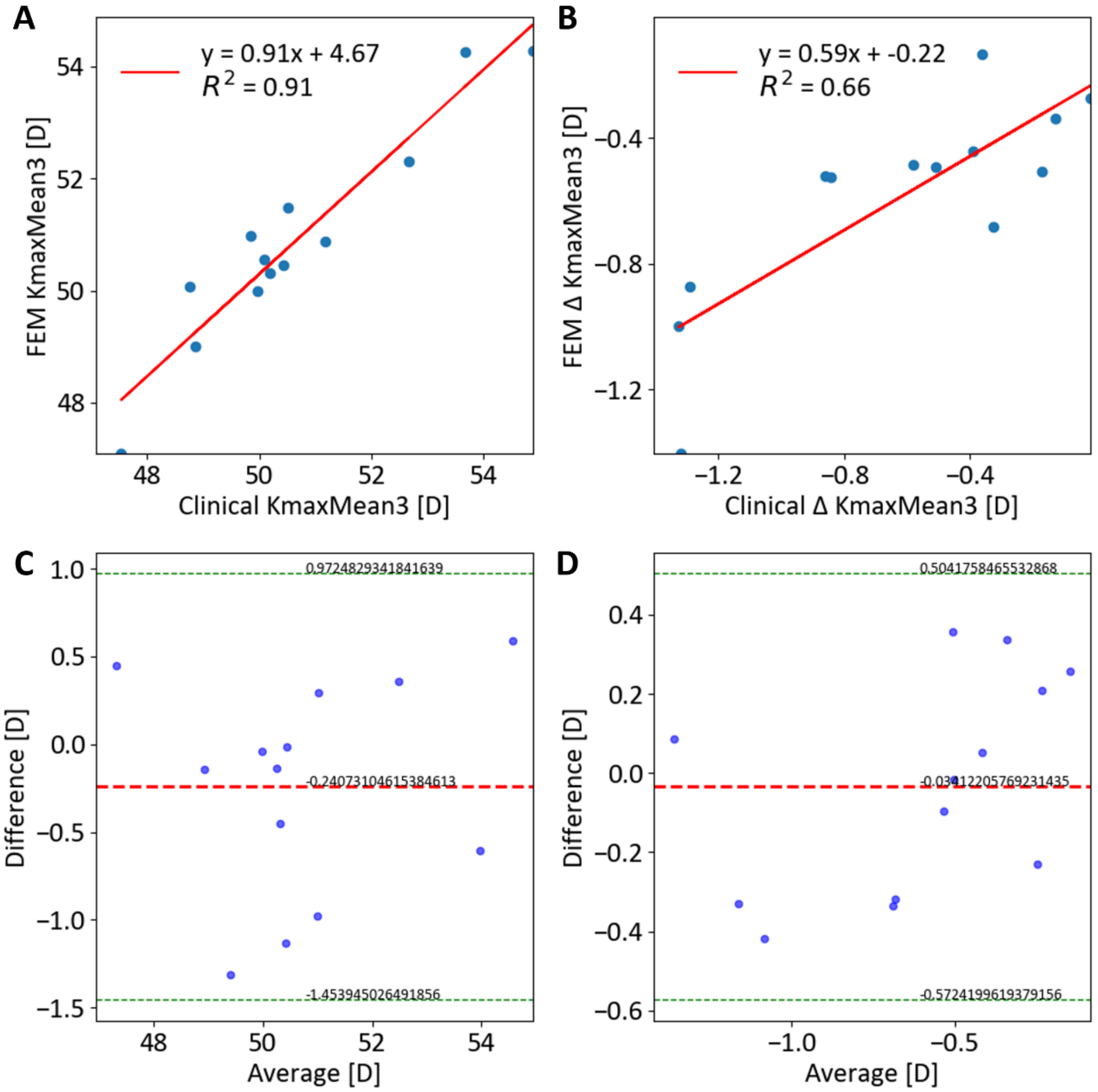

Figure S3: Comparison between clinical and FEM-simulated KmaxMean3 (left column) and Δ KmaxMean3 (right column) values after surgery for patients from cohort 1 who underwent corneal flattening at the 6 months follow-up (n=13). Correlation (top row) and Bland-Altman plot (bottom row).
